# False aneurysm of the radial artery: Unusual complication of both-bone forearm fracture in children: A case report

**DOI:** 10.1186/1757-1626-1-170

**Published:** 2008-09-19

**Authors:** A Amrani, MA Dandane, Z El Alami, T El Madhi, H Gourinda, A Miri

**Affiliations:** 1 16 allee safsaf, Amal II/01, Riyad, Rabat 10100, Morocco; 2 Paediatric orthopaedics' Unit, Children's hospital, Rabat 10100, Morocco

## Abstract

False aneurysm or pseudo aneurysm of an artery in close proximity to fractured bone is a well-recognized entity, and fewer various cases, involving different sites have been reported in the literature. We report new case of a Moroccan's patient who had 10-year-old boy presented with a right non displaced both-bone forearm fracture; the patient was placed in a long arm splint. After, six weeks, the cast was removed. And a pulsatile mass on the volar-radial aspect of the forearm was decouvred. The mass was non-tender and the patient had radial and ulnar pulse. An Ultrasound and brachial angiography showed a false aneurysm of distal radial and the radial artery was ligated.

In conclusion, pseudoaneurysm of the radial artery can be associated with any bone fracture despite non displacing fractures related to the elasticity of the bone in this age and orthopedic surgeons should be aware of this potential complication.

## Background

The most common causes of radial pseudo aneurysm in children and adolescents are penetrating trauma and iatrogenic arterial injury. False aneurysm or pseudo aneurysm of the artery in close proximity to the fractured bone is a well-recognized case, and a few other cases, involving different locations have been reported in literature [1-5]. We report the case of a patient with false aneurysm of the Radial Artery after a both-bone forearm fracture. As far as we know, only one such case had been previously reported in the literature [6].

Vascular complications following paediatric both bone forearm fractures are rare. This article presents a case of a non displaced both-bone forearm fracture in a child treated with a cast.

## Case report

**Figure 1:**
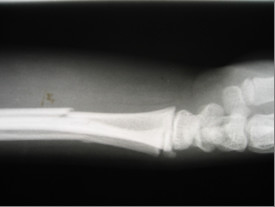
Plain radiographs of the forearm demonstrated a non-displaced both-bone radius and ulnar fracture.

A 10-year-old boy had a both-bone right forearm fracture from a fall with the outstretched hand. Examination revealed a closed injury with normal distal radial and ulnar pulses and intact sensation with no associated medical history or other clinical finding. Plain radiographs of the forearm (Figure 1) demonstrated a non-displaced both-bone radius and ulnar fracture and the patient's arm was placed in a long arm splint.

Six weeks later, the cast was removed. Examination demonstrated a 20-mm diameter fluid-filled pulsatile mass on the volar-radial side of the forearm approximately 4 cm from the wrist joint. The forearm and mass were non-tender and the patient had radial and ulnar pulse.

**Figure 2:**
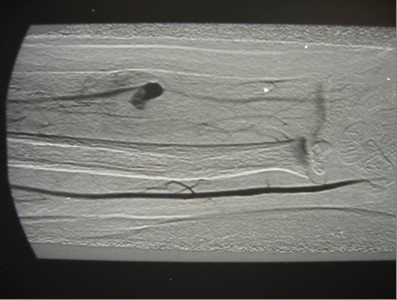
A brachial angiography show a false aneurysm of distal radial artery.

Radiographs demonstrated adequate alignment, a healing fracture and a good periosteal callus. Ultrasound was consistent with pseudoaneurysm of the right radial artery. Brachial angiography was performed to the patient. False aneurysm of distal radial artery was observed (Figure 2).

**Figure 3:**
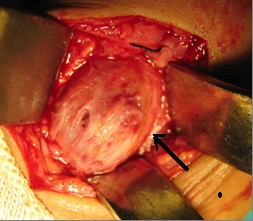
The mass was directly volar to the radial artery and extended into the subperiosteal of the radial sheath and the radial artery ligated.

A 4 cm longitudinal incision was made directly over the mass. The mass was directly volar to the radial artery and extended into the subperiosteal tissues. After the mass was freed of fibrous connections, it was excised, and the radial artery was ligated (Fig. 3). The patient did well postoperatively without any further complication. 3 years after surgery, the patient remains symptom free with normal growth and function of the hand.

## Discussion

The most common causes of radial pseudo aneurysm in children and adolescents are penetrating trauma and iatrogenic arterial injuries [7,8]. These vascular complications may occur within hours or months after the fracture. A pseudoaneurysm of an arterial vessel occurs after a full-thickness laceration from either blunt or penetrating trauma, from lacerations, or from complications arising from fracture fixation plates [1-3,6,9]. A vessel injury allows for extravasation of blood into the surrounding tissues with ensuing formation of a fibrous capsule. The artery usually remains patent due to the remaining inherent cylindrical structure of the artery, which prevents contraction and collapse. Thus the wall of the pseudoaneurysm is fibrous in nature and lacks an intima layer associated with native arteries. Blood is usually clotted to some degree within this fibrous wall and, in many cases; the entire mass will be pulstile owing to the patency of blood flow into the lumen of the pseudoaneurysm [5-9]. Despite a non-displaced bone fracture in our case, the occurrence of false aneurysm can be explained by the high degree of bone plasticity in this age and the artery was punctured by a fractured bone speculate before returning to its original position.

Operative treatment of pseudoaneurysms are not well defined. However, surgical treatment is recommended depending on their location, size, symptoms, and association with an important artery [6,8,10]. Observation has been recommended for asymptomatic pseudoaneurysms <10 mm in diameter, which involve arteries of only minor importance. 14 Pseudoaneurysms that are larger in size (>10 mm), located in an area that is predisposed to injury or associated with an important artery, should be operated surgically. Many surgical modalities have been described, including ligation or resection, direct repair, embolization or angioplasty, and vein grafting. The exact technique depends on location of the mass and the exact association with the vessel [6,13].

Other authors support simple ligation of radial artery and excision of the pseudo aneurysm if adequate collateral flow is observed. This treatment strategy is considered satisfactory even in the paediatric population [14,15]. We elected surgical treatment due to the fact that the patient was symptomatic, and then decided to ligate the radial artery since the vascular supply to the hand was assured by collateral circulation.

In summary, pseudoaneurysm of the radial artery can be associated with a forearm bone fracture despite non-displaced fractures related to the elasticity of the bone in this age and orthopaedic surgeons should be aware of this potential complication following a fracture of the bone forearm.

## Competing interests

The authors declare that they have no competing interests.

## Authors' contributions

AA: Operated, fellowed the case and wrote the manuscript. All others authors have read and improved the manuscript.

## Consent

A written informed consent was granted by the parents for publication of this case report and accompanying images. A copy of the written consent is available for the review of the Editor-in-Chief of this journal.
